# (3*E*,5*E*)-3,5-Bis(4-allyl­oxybenzyl­idene)-1-benzyl­piperidin-4-one

**DOI:** 10.1107/S1600536809046716

**Published:** 2009-11-11

**Authors:** N. S. Karthikeyan, K. Sathiyanarayanan, P. G. Aravindan, H. Ghosh, R. S. Rathore

**Affiliations:** a Chemistry Division, School of Science and Humanities, VIT University, Vellore 632014, India; b Physics Division, School of Science and Humanities, VIT University, Vellore 632014, India; c Bioinformatics Infrastructure Facility, Department of Biotechnology, School of Life Science, University of Hyderabad, Hyderabad 500 046, India

## Abstract

In the title compound C_32_H_31_NO_3_, the all­yloxy groups on either side of the piperidin-4-one ring are conformationally disordered. The contribution of major and minor components of the allyloxy group at the 3rd position of the ring are 0.576 (4) and 0.424 (4), respectively, and those at the 5th position are 0.885 (3) and 0.115 (3), respectively. The six-membered piperidin-4-one ring adopts a sofa conformation with the benzyl group occupying an equatorial position and the olefinic double bonds possessing an *E* configuration. Flanking phenyl substituents are stretched out on either side of the six-membered ring. π–π inter­actions with a centroid–centroid distance of 3.885 (1) Å give rise to mol­ecular dimers and short C—H⋯π contacts lead to chains along the *c* axis.

## Related literature

For related structures, see: Suresh *et al.* (2007 ▶); Rathore *et al.* (2009 ▶); Karthikeyan *et al.* (2009 ▶). For ring puckering parameters, see: Cremer & Pople (1975 ▶).
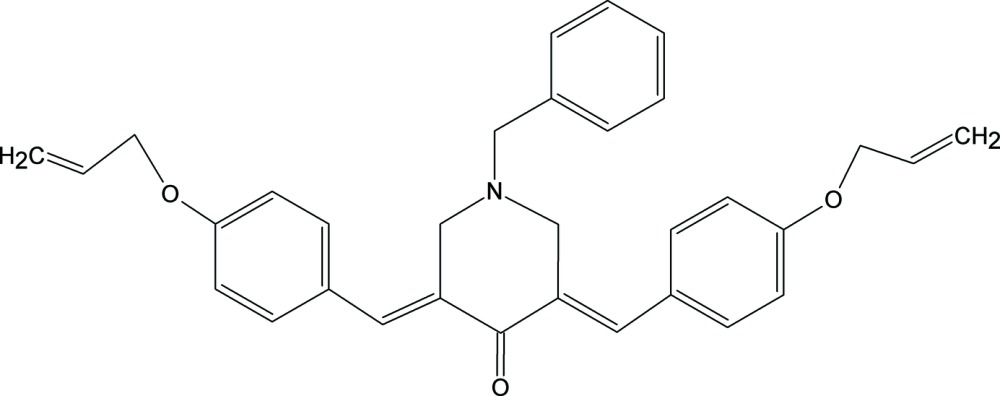


## Experimental

### 

#### Crystal data

C_32_H_31_NO_3_
*M* *_r_* = 477.58
Monoclinic, 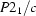

*a* = 15.6161 (4) Å
*b* = 9.2654 (2) Å
*c* = 18.9696 (5) Å
β = 111.031 (1)°
*V* = 2561.87 (11) Å^3^
*Z* = 4
Mo *K*α radiation
μ = 0.08 mm^−1^
*T* = 295 K
0.33 × 0.29 × 0.27 mm

#### Data collection

Bruker APEXII CCD area-detector diffractometer
Absorption correction: multi-scan (*SADABS*; Bruker, 2004 ▶) *T*_min_ = 0.896, *T*_max_ = 0.964
26042 measured reflections
5029 independent reflections
3882 reflections with *I* > 2σ(*I*)
*R*_int_ = 0.027

#### Refinement

*R*[*F* ^2^ > 2σ(*F* ^2^)] = 0.041
*wR*(*F* ^2^) = 0.114
*S* = 1.01
5029 reflections
342 parameters
1 restraint
H-atom parameters constrained
Δρ_max_ = 0.17 e Å^−3^
Δρ_min_ = −0.19 e Å^−3^

### 

Data collection: *APEX2* (Bruker, 2004 ▶); cell refinement: *SAINT-Plus* (Bruker, 2004 ▶); data reduction: *SAINT-Plus*; program(s) used to solve structure: *SHELXS97* (Sheldrick, 2008 ▶); program(s) used to refine structure: *SHELXL97* (Sheldrick, 2008 ▶); molecular graphics: *ORTEP-3* (Farrugia, 1997 ▶); software used to prepare material for publication: *SHELXL97*.

## Supplementary Material

Crystal structure: contains datablocks global, I. DOI: 10.1107/S1600536809046716/bq2170sup1.cif

Structure factors: contains datablocks I. DOI: 10.1107/S1600536809046716/bq2170Isup2.hkl

Additional supplementary materials:  crystallographic information; 3D view; checkCIF report

## Figures and Tables

**Table 1:** Hydrogen-bond geometry (Å, °)

*D*—H⋯*A*	*D*—H	H⋯*A*	*D*⋯*A*	*D*—H⋯*A*
C9—H9⋯*Cg*4^i^	0.93	2.97	3.8089 (19)	151
C33—H33*B*⋯*Cg*2^ii^	0.93	2.86	3.771 (4)	165

Symmetry codes: (i) 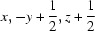; (ii) 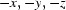. *Cg*2 is the centroid of the C8–C13 ring and *Cg*4 is the centroid of the C22–C27 ring.

**Fig. 1.**
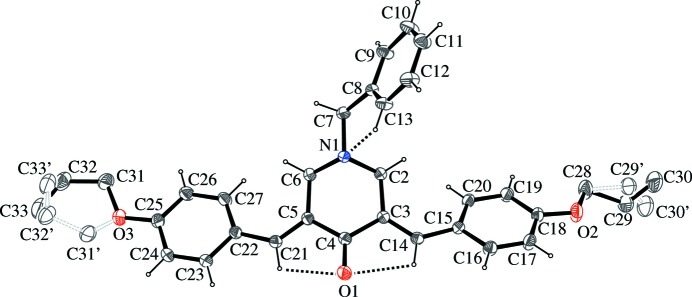
A view of (I) with non-H atoms shown as probability ellipsoids at 30% levels (Farrugia, 1997). H atoms radii are on an arbitrary scale and those connected to disordered components are omitted for clarity. The minor components of disordered group are shown in gray color. Dashed lines indicate intra-molecular interactions or bonds associated with disordered minor component.

**Fig. 2.**
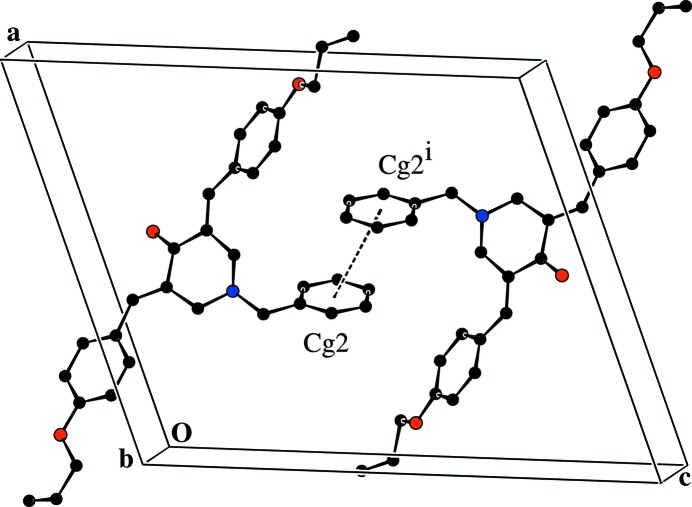
Molecular dimers viaπ-π interactions. Cg2 is the centroid of (C8—C13) ring. Symmetry code (i): 1 - x, -y, 1 - z.

**Fig. 3.**
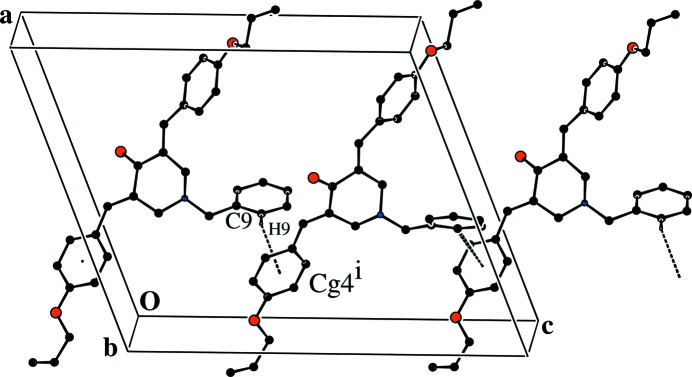
A one-dimensional chain via C—H···π short contact. (see Table 1 for symmetry code). Cg4 is the centroid of (C22—C27) ring.

## supplementary crystallographic information

### Comment

Derivatives of 3,5-diarylidene-4-piperidones (D4P) are pharmaceutically important compounds. We have synthesized a series of such compounds and investigated structures (Rathore *et al.*, 2009; Karthikeyan *et al.*, 2009). In continuation of this work, the title compound (3E,5E)-1-benzyl-3,5-bis(4-allyloxybenzylidene)piperidin-4-one, (I), has been synthesized and discussed here.

The structure of (I) with adopted atom-numbering scheme is shown in Fig 1. Phenyl substituents at the 3rd and 5t h positions of the piperidinone ring (also called piperidone; atoms N1/C2—C6) are stretched out on either side. The characteristic extended structure of flanking phenyl substituents is a common observation among previously reported analogous compounds: (*R*)-3,5-bis[(*E*)-benzylidene]-1-(1-phenylethyl)piperidin-4-one, 3,5-bis[(*E*)-4-chlorobenzylidene]-1-[(*R*)-1-phenylethyl]piperidin-4-one, and 3,5-bis[(*E*)-2-chlorobenzylidene]-1-[(*R*)-1-phenylethyl]piperidin-4-one (Suresh *et al.*, 2007), (3E,5E)-1-benzyl-3,5-bis(2-fluorobenzylidene)piperidin-4-one (Rathore *et al.*, 2009) and (3E,5E)-1-benzyl-3,5-dibenzylidenepiperidin-4-one (Karthikeyan *et al.*, 2009). The related torsion angles corresponding to phenyl substituents in (I) are as follows: C4—C3—C14—C15 = 173.76 (13)°, C3—C14—C15—C16 = 156.02 (15)°, C4—C5—C21—C22 = -179.52 (14)° and C5—C21—C22—C23 = -163.22 (16)°. C3, C5 olefinic double bonds have E configuration. The aromatic rings (C15—C20; C22—C27) of the phenyl substituents are significantly non-coplanar having an interplanar angle of 21.8 (1)°. The corresponding values are 3.29 (7)° and 41.2 (1)° in 3E,5E)-1-benzyl-3,5-bis(2-fluorobenzylidene)piperidin-4-one and (3E,5E)-1-benzyl-3,5-dibenzylidenepiperidin-4-one), respectively (Rathore *et al.*, 2009; Karthikeyan *et al.*, 2009).

In the piperidinone ring, the *sp*^2^ hybridized C3/C4/C5 leads to a low-energy sofa conformation of the six-membered ring, as also observed in the structures of related compounds (Suresh *et al.*, 2007; Rathore *et al.*, 2009; Karthikeyan *et al.*, 2009). In the sofa conformation, the N1 atom is -0.779 (1)Å shifted out of the base plane (C2/C3/C4/C5/C6). Ring puckering parameters (Cremer & Pople, 1975) for the atomic sequence (N1/C2/C3/C4/C5/C6) are as follows: q2 = 0.5544 (15) Å, q3 = 0.2301 (15) Å, φ = 5.60 (16)°, θ = 67.46 (14)°, and the total puckering amplitude Q = 0.6002 (15) Å. The benzyl group (C7—C13) at the 1st position is disposed towards C3-substituent and occupies an equatorial position of the piperidinone ring. The related torsion angles, describing its conformation are as follows: C2—N1—C7—C8 = -72.97 (16)°, N1—C7—C8—C9 = 156.00 (14)°.

The geometric parameters for observed short contacts are listed in Table 1. The characteristic intra-molecular interaction scheme, involving carbonyl and amino groups of piperidinone (Rathore *et al.*, 2009; Karthikeyan *et al.*, 2009) is also observed in (I). H14 and H21 participate in a contiguous intra-molecular C14—H14···O1···H21—C21 interaction scheme and proton H13 of C1-benzyl substituent participate in C13—H13···N1 (Fig. 1). Significant π-π interactions were observed. *Cg*2 (the centroid of C8—C13 ring) makes a parallel π-π interaction with *Cg*2^ii^ of inversion-related molecule [symmetry code(ii): 1 - *x*, -*y*, 1 - *z*] with a centroid-centroid distance of 3.885 (1) Å, a perpendicular distance of 3.5241 (8) Å, and a slippage of 1.635 Å. The π-π interaction between molecules gives rise to a molecular dimer (Fig 2). Packing is also characterized by short intermolecular C—H···O and C—H···π contacts (Table 1). Notably among them is C9—H9···*Cg*4^i^ that generate a linear chain parallel to *c* axis as shown in Fig. 3. *Cg*4 is the centroid of (C22—C27) ring.

### Experimental

A mixture of 1-benzyl-4-piperidone (0.01 mol) and 4-allyloxybenzaldehyde (0.02 mol) was added to a warm solution of ammonium acetate (0.01 mol) in absolute ethanol (15 ml). The mixture was gradually warmed on a water bath until the yellow color changed to orange. The mixture was kept aside overnight at room temperature. Reactions were monitored with TLC for completeness. The solid obtained was separated and the crude compound was purified using silica gel column chromatography with hexane and ethyl acetate as eluant. Final yields: 90.80%; m.p. 388 (1)°K. Suitable single crystals for data collection were grown from ethanol and tetrahydrofuran mixture.

### Refinement

The *p*-allyloxy groups (O2/C28—C30) and (O3/C31—C33) exhibit conformational disorder. The contribution of major and minor components are as follows: allyloxy group in the phenyl substituent at the 3rd position - (C28/C29/C30), 0.576 (4), (C28/C29'/C30'), 0.424 (4); those at 5t h position - (C31/C32/C33), 0.885 (3), (C31'/C32'/C33'), 0.115 (3). The corresponding atoms in the disordered groups were assigned the same equivalent isotropic displacement parameter. The reflection (1,0,0) was omitted as it was affected by extinction or absorption. Hydrogen atoms were placed in their stereochemically expected positions and refined with the riding options. The distances with hydrogen atoms are: C(aromatic/*sp*^2^)—H = 0.93 Å, *C*(methylene)—H = 0.97 Å, and *U*_iso_ = 1.2 *U*_eq_(parent).

### Crystal data

**Table:**

C_32_H_31_NO_3_	*F*(000) = 1016
*M**_r_* = 477.58	*D*_x_ = 1.238 Mg m^−^^3^
Monoclinic, *P*2_1_/*c*	Melting point: 388(1) K
Hall symbol: -P 2ybc	Mo *K*α radiation, λ = 0.71073 Å
*a* = 15.6161 (4) Å	Cell parameters from 9748 reflections
*b* = 9.2654 (2) Å	θ = 2.2–28.2°
*c* = 18.9696 (5) Å	µ = 0.08 mm^−^^1^
β = 111.031 (1)°	*T* = 295 K
*V* = 2561.87 (11) Å^3^	Block, yellow
*Z* = 4	0.33 × 0.29 × 0.27 mm

### Data collection

**Table:**

Bruker APEXII CCD area-detector diffractometer	5029 independent reflections
Radiation source: fine-focus sealed tube	3882 reflections with *I* > 2σ(*I*)
graphite	*R*_int_ = 0.027
φ and ω scans	θ_max_ = 26.0°, θ_min_ = 2.2°
Absorption correction: multi-scan (*SADABS*; Bruker, 2004)	*h* = −19→19
*T*_min_ = 0.896, *T*_max_ = 0.964	*k* = −11→11
26042 measured reflections	*l* = −21→23

### Refinement

**Table:**

Refinement on *F*^2^	Primary atom site location: structure-invariant direct methods
Least-squares matrix: full	Secondary atom site location: difference Fourier map
*R*[*F*^2^ > 2σ(*F*^2^)] = 0.041	Hydrogen site location: inferred from neighbouring sites
*wR*(*F*^2^) = 0.114	H-atom parameters constrained
*S* = 1.01	*w* = 1/[σ^2^(*F*_o_^2^) + (0.0509*P*)^2^ + 0.7272*P*] where *P* = (*F*_o_^2^ + 2*F*_c_^2^)/3
5029 reflections	(Δ/σ)_max_ = 0.001
342 parameters	Δρ_max_ = 0.17 e Å^−^^3^
1 restraint	Δρ_min_ = −0.19 e Å^−^^3^

### Special details

**Table:**

Geometry. All e.s.d.'s (except the e.s.d. in the dihedral angle between two l.s. planes) are estimated using the full covariance matrix. The cell e.s.d.'s are taken into account individually in the estimation of e.s.d.'s in distances, angles and torsion angles; correlations between e.s.d.'s in cell parameters are only used when they are defined by crystal symmetry. An approximate (isotropic) treatment of cell e.s.d.'s is used for============================================================================Weighted least-squares planes through the starred atoms (Nardelli, Musatti, Domiano & Andreetti Ric.Sci.(1965),15(II—A),807). Equation of the plane: m1**X*+m2*Y+m3**Z*=dPlane 1 m1 = 0.63145(0.00057) m2 = 0.53630(0.00051) m3 = -0.56005(0.00052) D = 2.64782(0.00814) Atom d s d/s (d/s)**2 C15 * 0.0025 0.0014 1.710 2.924 C16 * 0.0037 0.0016 2.339 5.471 C17 * -0.0099 0.0017 - 5.814 33.804 C18 * 0.0074 0.0016 4.481 20.083 C19 * -0.0002 0.0016 - 0.120 0.014 C20 * -0.0048 0.0016 - 3.021 9.124 O2 0.0675 0.0013 52.104 2714.813 C14 0.1183 0.0014 81.779 6687.801 C28 0.2295 0.0022 106.646 11373.305 ============ Sum((d/s)**2) for starred atoms 71.420 Chi-squared at 95% for 3 degrees of freedom: 7.81 The group of atoms deviates significantly from planarityPlane 2 m1 = 0.56481(0.00057) m2 = 0.77772(0.00043) m3 = -0.27594(0.00064) D = 3.26621(0.00176) Atom d s d/s (d/s)**2 C22 * -0.0163 0.0015 - 10.629 112.976 C23 * 0.0112 0.0016 6.834 46.705 C24 * 0.0078 0.0018 4.420 19.537 C25 * -0.0177 0.0017 - 10.604 112.437 C26 * 0.0113 0.0018 6.209 38.547 C27 * 0.0095 0.0017 5.532 30.601 O3 - 0.0627 0.0014 - 46.013 2117.186 C21 - 0.0323 0.0015 - 21.820 476.128 C31' -0.5200 0.0188 - 27.643 764.147 ============ Sum((d/s)**2) for starred atoms 360.801 Chi-squared at 95% for 3 degrees of freedom: 7.81 The group of atoms deviates significantly from planarityPlane 3 m1 = 0.32339(0.00075) m2 = 0.92133(0.00036) m3 = -0.21581(0.00070) D = 1.73547(0.00401) Atom d s d/s (d/s)**2 C2 * 0.0298 0.0015 20.137 405.483 C3 * -0.0224 0.0013 - 16.855 284.080 C5 * 0.0224 0.0013 16.863 284.370 C6 * -0.0338 0.0016 - 21.430 459.250 N1 - 0.7279 0.0012 - 601.376 361653.344 C4 - 0.1964 0.0014 - 139.920 19577.652 O1 - 0.4421 0.0012 - 370.110 136981.719 ============ Sum((d/s)**2) for starred atoms 1433.183 Chi-squared at 95% for 1 degrees of freedom: 3.84 The group of atoms deviates significantly from planarityPlane 4 m1 = 0.36687(0.00061) m2 = 0.89674(0.00032) m3 = -0.24754(0.00062) D = 1.84491(0.00391) Atom d s d/s (d/s)**2 C2 * 0.0029 0.0015 1.982 3.929 C3 * 0.0394 0.0013 29.495 869.956 C4 * -0.0904 0.0014 - 64.198 4121.393 C5 * 0.0833 0.0013 62.434 3897.966 C6 * -0.0612 0.0016 - 38.795 1505.066 N1 - 0.7792 0.0012 - 642.939 413370.656 O1 - 0.2648 0.0012 - 222.229 49385.770 ============ Sum((d/s)**2) for starred atoms 10398.310 Chi-squared at 95% for 2 degrees of freedom: 5.99 The group of atoms deviates significantly from planarityPlane 5 m1 = -0.85377(0.00041) m2 = -0.38211(0.00073) m3 = -0.35365(0.00075) D = -5.12945(0.00494) Atom d s d/s (d/s)**2 C8 * 0.0012 0.0015 0.816 0.665 C9 * -0.0076 0.0019 - 4.009 16.075 C10 * 0.0086 0.0022 3.946 15.574 C11 * 0.0003 0.0021 0.154 0.024 C12 * -0.0068 0.0020 - 3.356 11.260 C13 * 0.0041 0.0018 2.346 5.506 C7 0.0968 0.0016 59.458 3535.268 N1 - 0.4392 0.0013 - 345.718 119520.734 ============ Sum((d/s)**2) for starred atoms 49.105 Chi-squared at 95% for 3 degrees of freedom: 7.81 The group of atoms deviates significantly from planarityDihedral angles formed by LSQ-planes Plane - plane angle (s.u.) angle (s.u.) 1 2 21.83 (0.05) 158.17 (0.05) 1 3 35.00 (0.05) 145.00 (0.05) 1 4 31.66 (0.04) 148.34 (0.04) 1 5 56.91 (0.05) 123.09 (0.05) 2 3 16.52 (0.05) 163.48 (0.05) 2 4 13.36 (0.04) 166.64 (0.04) 2 5 47.02 (0.05) 132.98 (0.05) 3 4 3.39 (0.05) 176.61 (0.05) 3 5 56.51 (0.06) 123.49 (0.06) 4 5 55.37 (0.06) 124.63 (0.06)Ring puckering coordinates following Cremer D. & Pople J.A., JACS (1975).97,1354Ring 1 Atom Internal Cartesian coordinates *X* Y *Z* N1 0.0000(0.0000) 1.2939(0.0013) 0.4125(0.0009) C2 1.1736(0.0015) 0.7782(0.0017) -0.2803(0.0009) C3 1.2761(0.0013) -0.6993(0.0017) -0.0383(0.0009) C4 0.0068(0.0016) -1.4276(0.0014) 0.2246(0.0009) C5 - 1.2588(0.0015) -0.7183(0.0019) -0.0924(0.0010) C6 - 1.1977(0.0016) 0.7731(0.0016) -0.2262(0.0010) q2 = 0.5544(0.0013) q3 = 0.2301(0.0014) phi2 = 5.60 (0.14) Total puckering amplitude: QT = 0.6002(0.0013) Spherical polar angles: Theta2 = 67.46 (0.13)Asymmetry parameters Following Nardelli *M*., Acta Cryst.(1983). C39, 1141N1 C2 C3 C4 C5 C6DS(N1) =0.0379(0.0007) DS(N1 –C6) =0.2957(0.0006) D2(N1) =0.2492(0.0004) D2(N1 –C6) =0.1873(0.0006)DS(C2) =0.3212(0.0007) DS(C2 –N1) =0.3235(0.0006) D2(C2) =0.1726(0.0004) D2(C2 –N1) =0.1338(0.0006)DS(C3) =0.3591(0.0007) DS(C3 –C2) =0.1405(0.0006) D2(C3) =0.1453(0.0005) D2(C3 –C2) =0.3206(0.0006)============================================================================
Refinement. Refinement of *F*^2^ against ALL reflections. The weighted *R*-factor *wR* and goodness of fit *S* are based on *F*^2^, conventional *R*-factors *R* are based on *F*, with *F* set to zero for negative *F*^2^. The threshold expression of *F*^2^ > σ(*F*^2^) is used only for calculating *R*-factors(gt) *etc*. and is not relevant to the choice of reflections for refinement. *R*-factors based on *F*^2^ are statistically about twice as large as those based on *F*, and *R*- factors based on ALL data will be even larger.

### Fractional atomic coordinates and isotropic or equivalent isotropic displacement parameters (Å^2^)

**Table:**

	*x*	*y*	*z*	*U*_iso_*/*U*_eq_	Occ. (<1)
N1	0.39705 (8)	0.04800 (13)	0.23368 (6)	0.0381 (3)	
O1	0.53263 (7)	−0.08595 (13)	0.11005 (6)	0.0554 (3)	
O2	0.92659 (8)	0.17546 (14)	0.51308 (7)	0.0697 (4)	
O3	0.02843 (8)	0.17577 (15)	−0.19313 (7)	0.0667 (4)	
C2	0.49054 (9)	0.10326 (16)	0.26458 (8)	0.0386 (3)	
H2A	0.5174	0.0808	0.3180	0.046*	
H2B	0.4902	0.2073	0.2588	0.046*	
C3	0.54621 (9)	0.03515 (14)	0.22339 (8)	0.0345 (3)	
C4	0.49587 (9)	−0.01131 (15)	0.14423 (8)	0.0374 (3)	
C5	0.40104 (9)	0.04415 (14)	0.10699 (8)	0.0361 (3)	
C6	0.35317 (10)	0.10116 (17)	0.15698 (8)	0.0420 (3)	
H6A	0.3548	0.2058	0.1571	0.050*	
H6B	0.2894	0.0711	0.1375	0.050*	
C7	0.34420 (10)	0.08969 (18)	0.28031 (8)	0.0450 (4)	
H7A	0.2797	0.0732	0.2518	0.054*	
H7B	0.3524	0.1923	0.2907	0.054*	
C8	0.37001 (9)	0.01014 (15)	0.35407 (8)	0.0376 (3)	
C9	0.35206 (12)	0.07273 (18)	0.41302 (9)	0.0533 (4)	
H9	0.3287	0.1661	0.4079	0.064*	
C10	0.36824 (14)	−0.0012 (2)	0.47958 (10)	0.0635 (5)	
H10	0.3543	0.0415	0.5185	0.076*	
C11	0.40457 (13)	−0.1368 (2)	0.48862 (10)	0.0592 (5)	
H11	0.4162	−0.1863	0.5338	0.071*	
C12	0.42362 (13)	−0.19922 (18)	0.43080 (10)	0.0572 (4)	
H12	0.4488	−0.2914	0.4368	0.069*	
C13	0.40589 (11)	−0.12702 (17)	0.36363 (9)	0.0488 (4)	
H13	0.4183	−0.1714	0.3244	0.059*	
C14	0.63643 (9)	0.01206 (15)	0.25111 (8)	0.0375 (3)	
H14	0.6587	−0.0404	0.2196	0.045*	
C15	0.70605 (9)	0.05548 (15)	0.32273 (8)	0.0375 (3)	
C16	0.78690 (10)	−0.02438 (16)	0.35081 (9)	0.0461 (4)	
H16	0.7929	−0.1072	0.3253	0.055*	
C17	0.85777 (11)	0.01583 (18)	0.41499 (9)	0.0513 (4)	
H17	0.9104	−0.0406	0.4333	0.062*	
C18	0.85064 (10)	0.14063 (18)	0.45245 (9)	0.0484 (4)	
C19	0.77130 (10)	0.22157 (18)	0.42624 (9)	0.0498 (4)	
H19	0.7659	0.3048	0.4517	0.060*	
C20	0.70011 (10)	0.17874 (16)	0.36221 (9)	0.0439 (4)	
H20	0.6468	0.2338	0.3451	0.053*	
C21	0.36595 (10)	0.03912 (15)	0.03158 (8)	0.0398 (3)	
H21	0.4053	−0.0002	0.0098	0.048*	
C22	0.27741 (10)	0.08384 (16)	−0.02279 (8)	0.0395 (3)	
C23	0.25263 (10)	0.02939 (18)	−0.09592 (8)	0.0467 (4)	
H23	0.2931	−0.0313	−0.1075	0.056*	
C24	0.17044 (11)	0.06261 (19)	−0.15114 (9)	0.0520 (4)	
H24	0.1558	0.0242	−0.1993	0.062*	
C25	0.10945 (10)	0.15270 (18)	−0.13548 (9)	0.0492 (4)	
C26	0.13345 (12)	0.21327 (19)	−0.06456 (9)	0.0552 (4)	
H26	0.0938	0.2774	−0.0541	0.066*	
C27	0.21633 (11)	0.17866 (18)	−0.00919 (9)	0.0490 (4)	
H27	0.2316	0.2199	0.0384	0.059*	
C28	0.92820 (13)	0.3094 (2)	0.54962 (12)	0.0745 (6)	
H28A	0.8944	0.3026	0.5836	0.089*	0.576 (4)
H28B	0.9007	0.3844	0.5128	0.089*	0.576 (4)
H28C	0.8729	0.3207	0.5612	0.089*	0.424 (4)
H28D	0.9314	0.3883	0.5171	0.089*	0.424 (4)
C29	1.0299 (3)	0.3439 (6)	0.5941 (3)	0.0724 (11)	0.576 (4)
H29	1.0697	0.3548	0.5680	0.087*	0.576 (4)
C30	1.0601 (3)	0.3577 (6)	0.6636 (3)	0.0946 (13)	0.576 (4)
H30A	1.0211	0.3472	0.6905	0.114*	0.576 (4)
H30B	1.1218	0.3786	0.6889	0.114*	0.576 (4)
C29'	1.0091 (5)	0.3105 (9)	0.6193 (4)	0.0724 (11)	0.424 (4)
H29'	1.0120	0.2542	0.6607	0.087*	0.424 (4)
C30'	1.0778 (5)	0.3948 (9)	0.6207 (4)	0.0946 (13)	0.424 (4)
H30C	1.0734	0.4502	0.5787	0.114*	0.424 (4)
H30D	1.1305	0.3990	0.6638	0.114*	0.424 (4)
C31	−0.04398 (14)	0.2435 (3)	−0.17894 (12)	0.0689 (6)	0.885 (3)
H31A	−0.0326	0.3465	−0.1727	0.083*	0.885 (3)
H31B	−0.0485	0.2059	−0.1327	0.083*	0.885 (3)
C32	−0.13050 (19)	0.2170 (4)	−0.24256 (17)	0.0758 (8)	0.885 (3)
H32	−0.1830	0.2596	−0.2395	0.091*	0.885 (3)
C33	−0.1410 (2)	0.1413 (4)	−0.3016 (2)	0.0871 (9)	0.885 (3)
H33A	−0.0908	0.0963	−0.3074	0.105*	0.885 (3)
H33B	−0.1990	0.1314	−0.3386	0.105*	0.885 (3)
C31'	−0.0131 (11)	0.092 (2)	−0.2515 (10)	0.0689 (6)	0.115 (3)
H31C	0.0007	−0.0066	−0.2346	0.083*	0.115 (3)
H31D	0.0156	0.1098	−0.2884	0.083*	0.115 (3)
C32'	−0.1163 (18)	0.102 (3)	−0.2929 (19)	0.0758 (8)	0.115 (3)
H32'	−0.1481	0.0418	−0.3330	0.091*	0.115 (3)
C33'	−0.156 (2)	0.202 (4)	−0.267 (2)	0.0871 (9)	0.115 (3)
H33C	−0.1220	0.2602	−0.2272	0.105*	0.115 (3)
H33D	−0.2195	0.2158	−0.2895	0.105*	0.115 (3)

### Atomic displacement parameters (Å^2^)

**Table:**

	*U*^11^	*U*^22^	*U*^33^	*U*^12^	*U*^13^	*U*^23^
N1	0.0340 (6)	0.0505 (7)	0.0313 (6)	0.0005 (5)	0.0134 (5)	0.0023 (5)
O1	0.0495 (6)	0.0671 (7)	0.0453 (6)	0.0114 (5)	0.0118 (5)	−0.0158 (5)
O2	0.0484 (7)	0.0757 (8)	0.0625 (8)	0.0094 (6)	−0.0072 (6)	−0.0245 (6)
O3	0.0483 (7)	0.0974 (10)	0.0458 (7)	0.0175 (6)	0.0066 (5)	0.0048 (6)
C2	0.0375 (7)	0.0442 (8)	0.0335 (8)	−0.0022 (6)	0.0121 (6)	−0.0026 (6)
C3	0.0366 (7)	0.0324 (7)	0.0346 (7)	−0.0029 (5)	0.0128 (6)	0.0007 (5)
C4	0.0396 (7)	0.0360 (7)	0.0370 (8)	−0.0023 (6)	0.0143 (6)	−0.0011 (6)
C5	0.0368 (7)	0.0364 (7)	0.0342 (7)	−0.0038 (6)	0.0118 (6)	0.0004 (6)
C6	0.0385 (7)	0.0521 (9)	0.0344 (8)	0.0038 (6)	0.0118 (6)	0.0034 (6)
C7	0.0397 (8)	0.0576 (9)	0.0411 (8)	0.0087 (7)	0.0185 (7)	0.0065 (7)
C8	0.0347 (7)	0.0433 (8)	0.0372 (8)	−0.0004 (6)	0.0156 (6)	−0.0006 (6)
C9	0.0715 (11)	0.0474 (9)	0.0473 (10)	0.0133 (8)	0.0289 (8)	0.0008 (7)
C10	0.0885 (14)	0.0675 (11)	0.0449 (10)	0.0129 (10)	0.0363 (10)	0.0003 (8)
C11	0.0695 (11)	0.0650 (11)	0.0482 (10)	0.0087 (9)	0.0273 (9)	0.0151 (8)
C12	0.0716 (11)	0.0463 (9)	0.0647 (11)	0.0128 (8)	0.0376 (9)	0.0134 (8)
C13	0.0614 (10)	0.0464 (8)	0.0486 (9)	0.0050 (7)	0.0319 (8)	−0.0013 (7)
C14	0.0398 (7)	0.0359 (7)	0.0376 (8)	0.0004 (6)	0.0151 (6)	−0.0010 (6)
C15	0.0332 (7)	0.0416 (8)	0.0377 (8)	−0.0012 (6)	0.0128 (6)	−0.0003 (6)
C16	0.0428 (8)	0.0417 (8)	0.0509 (9)	0.0035 (6)	0.0132 (7)	−0.0063 (7)
C17	0.0388 (8)	0.0505 (9)	0.0554 (10)	0.0092 (7)	0.0057 (7)	−0.0026 (7)
C18	0.0382 (8)	0.0576 (9)	0.0423 (9)	0.0017 (7)	0.0057 (7)	−0.0063 (7)
C19	0.0429 (8)	0.0525 (9)	0.0495 (9)	0.0033 (7)	0.0112 (7)	−0.0142 (7)
C20	0.0338 (7)	0.0480 (8)	0.0463 (9)	0.0048 (6)	0.0102 (6)	−0.0052 (7)
C21	0.0392 (7)	0.0437 (8)	0.0379 (8)	−0.0025 (6)	0.0153 (6)	−0.0014 (6)
C22	0.0399 (8)	0.0454 (8)	0.0337 (8)	−0.0022 (6)	0.0137 (6)	0.0034 (6)
C23	0.0427 (8)	0.0614 (10)	0.0365 (8)	0.0041 (7)	0.0147 (7)	−0.0028 (7)
C24	0.0485 (9)	0.0724 (11)	0.0324 (8)	0.0028 (8)	0.0111 (7)	−0.0043 (7)
C25	0.0428 (8)	0.0632 (10)	0.0384 (9)	0.0052 (7)	0.0105 (7)	0.0082 (7)
C26	0.0563 (10)	0.0654 (10)	0.0438 (9)	0.0179 (8)	0.0177 (8)	0.0025 (8)
C27	0.0546 (9)	0.0551 (9)	0.0354 (8)	0.0067 (7)	0.0139 (7)	0.0002 (7)
C28	0.0606 (11)	0.0779 (13)	0.0653 (12)	0.0055 (10)	−0.0014 (9)	−0.0281 (10)
C29	0.055 (2)	0.092 (3)	0.065 (3)	−0.0181 (19)	0.0150 (16)	−0.025 (2)
C30	0.064 (2)	0.115 (3)	0.089 (4)	−0.017 (2)	0.007 (2)	−0.022 (3)
C29'	0.055 (2)	0.092 (3)	0.065 (3)	−0.0181 (19)	0.0150 (16)	−0.025 (2)
C30'	0.064 (2)	0.115 (3)	0.089 (4)	−0.017 (2)	0.007 (2)	−0.022 (3)
C31	0.0528 (12)	0.0854 (15)	0.0657 (14)	0.0211 (11)	0.0180 (10)	0.0095 (11)
C32	0.0505 (16)	0.0910 (18)	0.081 (2)	0.0139 (14)	0.0171 (13)	0.0238 (16)
C33	0.0545 (19)	0.106 (3)	0.088 (2)	−0.0036 (16)	0.0107 (18)	0.0102 (19)
C31'	0.0528 (12)	0.0854 (15)	0.0657 (14)	0.0211 (11)	0.0180 (10)	0.0095 (11)
C32'	0.0505 (16)	0.0910 (18)	0.081 (2)	0.0139 (14)	0.0171 (13)	0.0238 (16)
C33'	0.0545 (19)	0.106 (3)	0.088 (2)	−0.0036 (16)	0.0107 (18)	0.0102 (19)

### Geometric parameters (Å, °)

**Table:**

N1—C6	1.4538 (18)	C19—H19	0.9300
N1—C2	1.4570 (17)	C20—H20	0.9300
N1—C7	1.4616 (18)	C21—C22	1.457 (2)
O1—C4	1.2228 (17)	C21—H21	0.9300
O2—C18	1.3618 (18)	C22—C27	1.387 (2)
O2—C28	1.417 (2)	C22—C23	1.394 (2)
O3—C31'	1.314 (19)	C23—C24	1.369 (2)
O3—C25	1.3599 (18)	C23—H23	0.9300
O3—C31	1.401 (2)	C24—C25	1.377 (2)
C2—C3	1.5006 (19)	C24—H24	0.9300
C2—H2A	0.9700	C25—C26	1.380 (2)
C2—H2B	0.9700	C26—C27	1.380 (2)
C3—C14	1.3324 (19)	C26—H26	0.9300
C3—C4	1.4869 (19)	C27—H27	0.9300
C4—C5	1.4851 (19)	C28—C29'	1.465 (8)
C5—C21	1.3365 (19)	C28—C29	1.540 (5)
C5—C6	1.4986 (19)	C28—H28A	0.9700
C6—H6A	0.9700	C28—H28B	0.9700
C6—H6B	0.9700	C28—H28C	0.9700
C7—C8	1.503 (2)	C28—H28D	0.9700
C7—H7A	0.9700	C29—C30	1.237 (8)
C7—H7B	0.9700	C29—H29	0.9300
C8—C13	1.374 (2)	C30—H30A	0.9300
C8—C9	1.375 (2)	C30—H30B	0.9300
C9—C10	1.378 (2)	C29'—C30'	1.319 (11)
C9—H9	0.9300	C29'—H29'	0.9300
C10—C11	1.364 (2)	C30'—H30C	0.9300
C10—H10	0.9300	C30'—H30D	0.9300
C11—C12	1.364 (2)	C31—C32	1.474 (3)
C11—H11	0.9300	C31—H31A	0.9700
C12—C13	1.376 (2)	C31—H31B	0.9700
C12—H12	0.9300	C32—C33	1.280 (4)
C13—H13	0.9300	C32—H32	0.9300
C14—C15	1.4593 (19)	C33—H33A	0.9300
C14—H14	0.9300	C33—H33B	0.9300
C15—C20	1.387 (2)	C31'—C32'	1.52 (3)
C15—C16	1.394 (2)	C31'—H31C	0.9700
C16—C17	1.371 (2)	C31'—H31D	0.9700
C16—H16	0.9300	C32'—C33'	1.307 (10)
C17—C18	1.382 (2)	C32'—H32'	0.9300
C17—H17	0.9300	C33'—H33C	0.9300
C18—C19	1.379 (2)	C33'—H33D	0.9300
C19—C20	1.378 (2)		
			
C6—N1—C2	109.14 (11)	C19—C20—H20	119.2
C6—N1—C7	110.51 (11)	C15—C20—H20	119.2
C2—N1—C7	111.44 (11)	C5—C21—C22	131.92 (14)
C18—O2—C28	118.54 (13)	C5—C21—H21	114.0
C31'—O3—C25	128.3 (7)	C22—C21—H21	114.0
C31'—O3—C31	103.7 (7)	C27—C22—C23	116.76 (14)
C25—O3—C31	119.87 (14)	C27—C22—C21	126.12 (13)
N1—C2—C3	109.08 (11)	C23—C22—C21	117.09 (13)
N1—C2—H2A	109.9	C24—C23—C22	121.95 (15)
C3—C2—H2A	109.9	C24—C23—H23	119.0
N1—C2—H2B	109.9	C22—C23—H23	119.0
C3—C2—H2B	109.9	C23—C24—C25	120.16 (15)
H2A—C2—H2B	108.3	C23—C24—H24	119.9
C14—C3—C4	116.93 (12)	C25—C24—H24	119.9
C14—C3—C2	126.17 (13)	O3—C25—C24	115.97 (14)
C4—C3—C2	116.90 (11)	O3—C25—C26	124.68 (15)
O1—C4—C5	121.41 (13)	C24—C25—C26	119.36 (14)
O1—C4—C3	121.37 (13)	C25—C26—C27	119.99 (15)
C5—C4—C3	117.11 (12)	C25—C26—H26	120.0
C21—C5—C4	116.93 (13)	C27—C26—H26	120.0
C21—C5—C6	125.70 (13)	C26—C27—C22	121.68 (15)
C4—C5—C6	117.37 (12)	C26—C27—H27	119.2
N1—C6—C5	110.54 (12)	C22—C27—H27	119.2
N1—C6—H6A	109.5	O2—C28—C29'	107.8 (3)
C5—C6—H6A	109.5	O2—C28—C29	106.5 (2)
N1—C6—H6B	109.5	O2—C28—H28A	110.4
C5—C6—H6B	109.5	C29—C28—H28A	110.4
H6A—C6—H6B	108.1	O2—C28—H28B	110.4
N1—C7—C8	114.38 (12)	C29—C28—H28B	110.4
N1—C7—H7A	108.7	H28A—C28—H28B	108.6
C8—C7—H7A	108.7	O2—C28—H28C	110.1
N1—C7—H7B	108.7	C29'—C28—H28C	110.1
C8—C7—H7B	108.7	O2—C28—H28D	110.1
H7A—C7—H7B	107.6	C29'—C28—H28D	110.1
C13—C8—C9	118.37 (14)	H28C—C28—H28D	108.5
C13—C8—C7	122.46 (13)	C30—C29—C28	122.0 (5)
C9—C8—C7	119.05 (13)	C30—C29—H29	119.0
C8—C9—C10	120.77 (15)	C28—C29—H29	119.0
C8—C9—H9	119.6	C29—C30—H30A	120.0
C10—C9—H9	119.6	C29—C30—H30B	120.0
C11—C10—C9	120.30 (16)	H30A—C30—H30B	120.0
C11—C10—H10	119.8	C30'—C29'—C28	117.0 (7)
C9—C10—H10	119.8	C30'—C29'—H29'	121.5
C12—C11—C10	119.36 (16)	C28—C29'—H29'	121.5
C12—C11—H11	120.3	C29'—C30'—H30C	120.0
C10—C11—H11	120.3	C29'—C30'—H30D	120.0
C11—C12—C13	120.60 (15)	H30C—C30'—H30D	120.0
C11—C12—H12	119.7	O3—C31—C32	109.5 (2)
C13—C12—H12	119.7	O3—C31—H31A	109.8
C8—C13—C12	120.57 (14)	C32—C31—H31A	109.8
C8—C13—H13	119.7	O3—C31—H31B	109.8
C12—C13—H13	119.7	C32—C31—H31B	109.8
C3—C14—C15	130.70 (13)	H31A—C31—H31B	108.2
C3—C14—H14	114.6	C33—C32—C31	126.9 (3)
C15—C14—H14	114.6	C33—C32—H32	116.6
C20—C15—C16	117.24 (13)	C31—C32—H32	116.6
C20—C15—C14	123.90 (13)	C32—C33—H33A	120.0
C16—C15—C14	118.66 (13)	C32—C33—H33B	120.0
C17—C16—C15	121.81 (14)	H33A—C33—H33B	120.0
C17—C16—H16	119.1	O3—C31'—C32'	120.8 (17)
C15—C16—H16	119.1	O3—C31'—H31C	107.1
C16—C17—C18	119.71 (14)	C32'—C31'—H31C	107.1
C16—C17—H17	120.1	O3—C31'—H31D	107.1
C18—C17—H17	120.1	C32'—C31'—H31D	107.1
O2—C18—C19	124.96 (14)	H31C—C31'—H31D	106.8
O2—C18—C17	115.21 (14)	C33'—C32'—C31'	114 (3)
C19—C18—C17	119.81 (14)	C33'—C32'—H32'	122.9
C20—C19—C18	119.82 (14)	C31'—C32'—H32'	122.9
C20—C19—H19	120.1	C32'—C33'—H33C	120.0
C18—C19—H19	120.1	C32'—C33'—H33D	120.0
C19—C20—C15	121.59 (14)	H33C—C33'—H33D	120.0
			
C6—N1—C2—C3	−68.12 (14)	C16—C17—C18—O2	−176.47 (16)
C7—N1—C2—C3	169.55 (11)	C16—C17—C18—C19	1.9 (3)
N1—C2—C3—C14	−152.11 (13)	O2—C18—C19—C20	177.17 (16)
N1—C2—C3—C4	27.71 (16)	C17—C18—C19—C20	−1.0 (3)
C14—C3—C4—O1	10.5 (2)	C18—C19—C20—C15	−0.2 (2)
C2—C3—C4—O1	−169.32 (13)	C16—C15—C20—C19	0.5 (2)
C14—C3—C4—C5	−165.87 (12)	C14—C15—C20—C19	−174.31 (15)
C2—C3—C4—C5	14.30 (18)	C4—C5—C21—C22	−179.52 (14)
O1—C4—C5—C21	−15.5 (2)	C6—C5—C21—C22	0.3 (3)
C3—C4—C5—C21	160.86 (12)	C5—C21—C22—C27	18.6 (3)
O1—C4—C5—C6	164.67 (14)	C5—C21—C22—C23	−163.22 (16)
C3—C4—C5—C6	−18.95 (18)	C27—C22—C23—C24	−2.6 (2)
C2—N1—C6—C5	63.70 (15)	C21—C22—C23—C24	178.99 (15)
C7—N1—C6—C5	−173.41 (12)	C22—C23—C24—C25	0.3 (3)
C21—C5—C6—N1	161.14 (13)	C31'—O3—C25—C24	24.9 (11)
C4—C5—C6—N1	−19.07 (18)	C31—O3—C25—C24	168.30 (18)
C6—N1—C7—C8	165.49 (12)	C31'—O3—C25—C26	−155.5 (11)
C2—N1—C7—C8	−72.97 (16)	C31—O3—C25—C26	−12.1 (3)
N1—C7—C8—C13	−28.1 (2)	C23—C24—C25—O3	−177.96 (15)
N1—C7—C8—C9	156.00 (14)	C23—C24—C25—C26	2.4 (3)
C13—C8—C9—C10	−1.1 (3)	O3—C25—C26—C27	177.74 (16)
C7—C8—C9—C10	175.03 (16)	C24—C25—C26—C27	−2.7 (3)
C8—C9—C10—C11	1.6 (3)	C25—C26—C27—C22	0.2 (3)
C9—C10—C11—C12	−0.8 (3)	C23—C22—C27—C26	2.4 (2)
C10—C11—C12—C13	−0.5 (3)	C21—C22—C27—C26	−179.42 (15)
C9—C8—C13—C12	−0.3 (2)	C18—O2—C28—C29'	170.8 (3)
C7—C8—C13—C12	−176.20 (15)	C18—O2—C28—C29	−159.7 (3)
C11—C12—C13—C8	1.0 (3)	O2—C28—C29—C30	−119.0 (5)
C4—C3—C14—C15	173.76 (13)	C29'—C28—C29—C30	−22.0 (7)
C2—C3—C14—C15	−6.4 (2)	O2—C28—C29'—C30'	108.0 (7)
C3—C14—C15—C20	−29.2 (2)	C29—C28—C29'—C30'	16.1 (6)
C3—C14—C15—C16	156.02 (15)	C31'—O3—C31—C32	−11.6 (9)
C20—C15—C16—C17	0.4 (2)	C25—O3—C31—C32	−162.8 (2)
C14—C15—C16—C17	175.49 (14)	O3—C31—C32—C33	1.7 (4)
C15—C16—C17—C18	−1.6 (3)	C25—O3—C31'—C32'	155.8 (16)
C28—O2—C18—C19	−4.7 (3)	C31—O3—C31'—C32'	8(2)
C28—O2—C18—C17	173.51 (17)	O3—C31'—C32'—C33'	3(4)

### Hydrogen-bond geometry (Å, °)

**Table:**

*D*—H···*A*	*D*—H	H···*A*	*D*···*A*	*D*—H···*A*
C13—H13···N1	0.93	2.61	2.912 (2)	100
C14—H14···O1	0.93	2.33	2.7385 (18)	106
C21—H21···O1	0.93	2.34	2.7482 (19)	106
C9—H9···Cg4^i^	0.93	2.97	3.8089 (19)	151
C33—H33A···O3	0.93	2.41	2.726 (4)	100
C33—H33B···Cg2^ii^	0.93	2.86	3.771 (4)	165

Symmetry codes: (i) *x*, −*y*+1/2, *z*+1/2; (ii) −*x*, −*y*, −*z*.
